# The impact of personal health literacy and school health literacy environments on schoolteachers' health outcomes

**DOI:** 10.3389/fpubh.2025.1570615

**Published:** 2025-05-15

**Authors:** Mingyang Yu, Rongmei Liu, Qiuping Zhao, Junfang Wang, Yuxi Bai, Xiaomo Yang, Shuaibin Liu, Orkan Okan, Bing Hua, Nengguang Dai, Suyan Xu, Shuaijun Guo

**Affiliations:** ^1^Hypertension Prevention and Treatment Centre of Henan Province, Fuwai Central China Cardiovascular Hospital, Zhengzhou, China; ^2^Community Health Centre of Chaohe, Zhengzhou, China; ^3^School of Medicine and Health, Technical University of Munich, Munich, Germany; ^4^Zhengzhou Community Health Association, Zhengzhou, China; ^5^Grassroots Health and Health Department, Health Commission of Henan Province, Zhengzhou, China; ^6^Department of Pharmacy, Fuwai Central China Cardiovascular Hospital, Zhengzhou, China; ^7^Department of Pharmacy, Henan Provincial People's Hospital, Zhengzhou, China; ^8^Centre for Community Child Health, Murdoch Children's Research Institute, Melbourne, VIC, Australia; ^9^Department of Pediatrics, University of Melbourne, Melbourne, VIC, Australia

**Keywords:** personal health literacy, organisational health literacy, health outcomes, teachers, HLS_19_-Q12

## Abstract

**Background:**

The relationship between personal health literacy and health outcomes is clear, but the role of health literacy environments is often overlooked. This study examined associations between personal health literacy, school health literacy environments and health outcomes among schoolteachers.

**Methods:**

A cross-sectional study was conducted among 7,364 schoolteachers in Zhengzhou, China. Personal health literacy was measured by the Health Literacy Population Survey 2019–2021 (excellent/sufficient/problematic/inadequate) and school health literacy environments were measured by the Organisational Health Literacy of School Questionnaire (supportive/less supportive). Health outcomes included health status (poor/good), health-compromising behaviours (yes/no), health service use (yes/no), and healthcare cost (≥RMB 1,000/ <RMB 1,000).

**Results:**

Over half of teachers had inadequate or problematic health literacy. Teachers with inadequate health literacy had higher odds of poor health status, health-compromising behaviour, health service use, and high healthcare cost than those with excellent health literacy. Similarly, teachers who perceived less supportive school health literacy environments had higher odds of poor health outcomes.

**Conclusion:**

Both personal health literacy and school health literacy environments are important to schoolteachers' health outcomes. Educational programs and organisational change are needed to improve personal health literacy and school environments to improve schoolteachers' health and wellbeing.

## 1 Introduction

Health literacy is a fundamental determinant of health (1). Extant literature has shown that low health literacy is associated with a range of negative health outcomes, including poor health status, health-compromising behaviours, more health service use, and high healthcare costs (2). In the global health promotion agenda from the World Health Organization (3), addressing low health literacy is a part of the strategy to address population health inequities. Many national governments and international organisations have integrated health literacy into their health agendas and initiatives and even developed national action plans to improve population health literacy and reduce health disparities (3, 4).

Health literacy is about how an individual manages health information in everyday life to make critical health judgments and inform healthy behaviours (5, 6). However, health literacy goes beyond the individual and should be understood as an interactive outcome between personal health skills and the broad environment in which an individual lives. The Australian Commission on Safety and Quality in Health Care defines health literacy as comprising two components (7): personal health literacy that represents one's ability to access, understand, appraise, and apply health information to maintain and improve personal health, and health literacy environments which refer to the systems and relationships that influence how health information is communicated.

Currently, there have been a number of studies that examine the measures, levels, influencing factors, and impact of personal health literacy across populations and contexts (2, 8, 9). Findings from the 2019–2021 European health literacy survey showed that low health literacy was prevalent across countries, ranging from 25% in Slovakia to 72% in Germany (9). In Southeast Asian countries, the prevalence of low health literacy ranged from 6.0% to 94.2% (10). In China, the 2021 national health literacy survey showed 65.9% of Chinese residents aged 15–69 years had low health literacy (11). However, most existing studies focus on personal health literacy in adults, neglecting the role of health literacy environments, which are an integral part of understanding health literacy in its fullest sense. This is more so true when it comes to children and settings relevant to their health and health literacy (12). Addressing this issue requires considering certain professionals working in school such as schoolteachers.

Schoolteachers play a crucial role in shaping the intellectual and emotional development of school-aged children, but also in the development of children's health literacy (13). They are uniquely positioned to deliver health education, equipping children with essential health knowledge, attitudes, and behaviours. In addition, they often engage with families and communities (12), creating a holistic approach to developing children's health literacy that extends beyond the classroom. Understanding schoolteachers' health literacy is of paramount importance as it not only influences their own health and wellbeing, but also influences the way they provide health education, thus impacting children's health literacy (13).

Schools are formal educational organisations and offer infrastructures, resources, and environments that can enable the success of health education (12), and in particular regarding school health literacy environments (14). The Health Promoting School (HPS) framework highlights five key action areas of health promotion in school settings (15): (i) building healthy public policy, (ii) creating supportive environments for health, (iii) strengthening community action for health, (iv) developing personal skills, and (v) re-orienting health services. Empirical evidence shows that all these components have a critical role in equipping both children's and schoolteachers' health literacy and fostering their health and wellbeing (16, 17). Understanding school health literacy environments is crucial for identifying possible ways to better support children's and schoolteachers' health and wellbeing.

Although it has been raised more than two decades (13), schoolteachers' health literacy has only gained increasing attention as part of school health promotion programs in the last decade (13, 18). Findings from previous national (19) and international studies (20–22) showed that schoolteachers' health literacy was generally low and there were disparities in health literacy by sociodemographics such as age, gender, and marital status. However, very few studies have examined the association between schoolteachers' health literacy and health outcomes (16). Furthermore, the role of school health literacy environments is often overlooked. To fill these gaps, the present study aimed to investigate the associations of personal health literacy and school health literacy environments with schoolteachers' health outcomes.

## 2 Materials and methods

### 2.1 Study design and settings

A cross-sectional study was designed to recruit teachers from 11 schools (five urban and six rural) in Zhengzhou, Henan Province, China, using convenience cluster sampling. In brief, two districts (Jingkai District/Zhongmou County) were selected according to their socioeconomic levels, one representing high and the other representing low. Based on previous successful collaborations with schools, we selected five or six schools in each district (Jingkai District: three primary schools and two middle schools; Zhongmou County: four primary schools and two middle schools). At each school, all teaching staff were invited to complete an online self-administered questionnaire via Wenjuanxing. Participants received written online information about the study in Chinese. Informed consent was obtained from all respondents prior to filling out the questionnaire. Based on previous prevalence studies in Chinese teachers (23) and sample size calculation formula [$n=\left(\frac{z^{2}\times p\left(1-p\right)}{e^{2}}\right)$], we estimated a sample size of at least 232 in each district (where *p* = 0.185, *z* = 1.96, *e* = 0.05). Considering the potential non-response rate of 30%, the final sample size should be at least 664. Data collection was undertaken between 20 September 2022 and 13 June 2023. We used the Strengthening the Reporting of Observational Studies in Epidemiology (STROBE) checklist as guidelines to ensure the reporting quality of the present study (see Supplementary Table S1).

### 2.2 The questionnaire

Data collected included key sociodemographics, personal health literacy, school health literacy environments, and health outcomes (see Table 1 for measurement details). In total, there were four parts in this questionnaire (Part 1: You and Your Family; Part 2: Your Health Literacy; Part 3: Your School; Part 4: Your Personal Health), with each part having 8–12 questions. The average time to complete the survey was 10 min.

**Table 1:** Measurement details of sociodemographics, personal health literacy, school health literacy environments and health outcomes.

**Variable**	**Measurement details**
Sociodemographics	We collected socio-demographic information on geographic location (Jingkai District/Zhongmou County), school type (primary/secondary), participants' sex (male/female), age group (30 years or below/31–39 years/40 years or above), ethnicity (Han/ethnic minorities), marital status (unmarried/married/divorced or widowed), highest educational level (Bachelor or above/Diploma or below), teaching duration (1–4 years/5–9 years/10–14 years/15–19 years/20–24 years/25 years or more), teaching subject (literacy/math/English/physics/chemistry/biology/history/geography/politics/physical education/music/art/health/other/more than one subject), chronic health conditions (yes/no), health awareness in daily life (very important/not very important), and medical insurance (medical insurance for urban workers/medical insurance for urban and rural residents/rural cooperative medical insurance/commercial medical insurance).
Personal health literacy	Personal health literacy was measured by the 12-item Chinese version of the European Health Literacy Population Survey 2019–2021 (HLS_19_-Q12) (9, 35), which comprising three health domains and four aspects of health information management. Respondents answered each item (e.g., “*On a scale from very easy to very difficult, how easy would you say it is: to find out where to get professional help when you are ill?”*) on a four-point Likert scale (1 = very difficult, 4 = very easy) concerning the experienced difficulty of each task. The Chinese version the HLS_19_-Q12 has shown excellent reliability and strong validity in the general population of Chinese adults (35). In the present study, our sample had Cronbach's alpha values of 0.92. The total score of the HLS_19_-Q12 was calculated as the percentage of items with valid responses that were answered with “very easy” or “easy” provided that at least 80% of the items contain valid responses (9). If <80% of the items contain valid responses, the score was coded as “missing.” Higher scores of the HLS_19_-Q12 indicate higher levels of health literacy. Four categories were created based on the recommended cut-off points (9): (i) Excellent: “very easy”≥50 and “very difficult” + “difficult” <8.334, the number of answers with “very easy” should be above $\frac{1}{2}$ and the answers for “very difficult” + “difficult” should be no more than 1/12; (ii) Sufficient: “very easy” + “easy”>83.33, at least 10 out of the 12 items should be answered with “very easy” or “easy” and not more than 2 out of 12 with “very difficult” or “difficult”; (iii) Problematic: all respondents who were not in groups of “excellent”, “sufficient”, or “inadequate”; and (iv) Inadequate: “very easy” <8.334, “very difficult” and “difficult”≥50, the number of answers with “very difficult” + “difficult” should be above $\frac{1}{2}$ and for “very easy” should be no more than 1/12.
School health literacy environments	School health literacy environments were assessed by the Short Form of the Organisational Health Literacy of School Questionnaire (OHLS-Q-SF) (14), which consists of eight items that measure processes and structures regarding the promotion of health literacy in school (36, 37). Participants answered each item (e.g., “*The design of everyday school life contributes to promoting health literacy at our school”*) on a four-point scale (1 = strongly agree, 2 = agree, 3 = disagree, 4 = strongly disagree). The OHLS-Q-SF showed high reliability (Cronbach's alpha = 0.97) and strong validity (comparative fit index = 0.985, Tucker and Lewis's index of fit = 0.978, root mean square error of approximatio*n =* 0.093 (95% CI = 0.088, 0.097) in our sample. The OHLS-Q-SF total score (ranging 0–32) was summed by reversing the code of each item, with higher scores indicating higher levels of school health literacy environments. In keeping with previous studies (38), we used the top 25% as a cut-off to indicate a supportive school health literacy environment.
Health status	Health status was assessed using a widely-used general self-report health question (‘In general, would you say your health is?' 1 = poor, 5 = excellent) (39). Poor health status was defined as “yes” if participants answered “poor” or “fair”.
Health-compromising behaviours	Health-compromising behaviours were measured by three items derived from previously well-established surveys (40), which included cigarette smoking [“Are you smoking?”; 1 = currently; 2 = ever; 3 = never), alcohol drinking (“Have you had any alcohol in the past 30 days? (more than half a bottle or a can of beer, a small cup of spirit, a glass of wine or yellow wine)”; 1 = yes; 2 = no), and physical inactivity (“How many times have you exercised for 30 min or more in the past 30 days, such as running, walking, cycling, etc.?”; 1 = almost every day; 2 = several times a week; 3 = several times a month; 4 = almost not at all). Each item was first dichotomised (cigarette smoking: yes = currently or ever/no = never); alcohol drinking: yes/no; physical inactivity: yes = several times a month or almost not at all/no = almost every day or several times a week)] and then a composite measure of health-compromising behaviours was created if participants had at least one health-compromising behaviour.
Health service use	Health service use was assessed by four items derived from previously well-established surveys (40), which included emergency service use (“How many times have you used the emergency service in the last 12 months?”), general practitioner service use (“How many times have you been to see a doctor in the last 12 months?”), hospitalisation (“How many times have you stayed in a hospital for treatment in the last 12 months?”), and patient-provider communication (“How many times have you raised a question during your doctor's appointment in the last 12 months?”). Participants answered each item on a four-point scale (1 = 0 times; 2 = 1–2 times; 3 = 3–5 times; 4 = 6 or more times). Each item was first dichotomised (yes=1–2 times or 3–5 times or 6 or more times)/no = 0 times) and then a composite measure of health service use was created if participants had used at least one service.
Healthcare cost	Healthcare cost was self-reported by participants about the amount of out-of-pocket health expenditure [“What was your out-of-pocket cost for healthcare (e.g., consultations, medicines, and tests) in the last year?”] (27). Participants were coded as having high healthcare cost if they spent RMB 1000 or more (27).

### 2.3 Statistical analysis

All statistical analyses were performed using Stata 18.0 (StataCorp, Texas, USA). Descriptive statistics were conducted to show the distribution of participants' characteristics, personal health literacy, school health literacy environments, and each health outcome. Univariate analysis was used to examine the relationship between participants' characteristics and levels of personal health literacy/school health literacy environments. Next, a series of logistic regression analyses were conducted to examine the associations between personal health literacy/school health literacy environments and each health outcome. Model 1 was unadjusted. Model 2 was adjusted for all covariates (i.e., geographic location, school type, sex, age group, ethnicity, marital status, highest educational level, teaching duration, teaching subject, chronic health conditions, health awareness in daily life, and medical insurance). Model 3 was additionally adjusted for school health literacy environments/personal health literacy when examining the association between personal health literacy/school health literacy environments and each health outcome.

### 2.4 Missing data

The proportion of respondents with complete data across all study variables was 89.9% (see Appendix 1 for details). To examine the potential impact of missing data, we used multiple imputation by chained equations to reduce the potential bias due to incomplete records (24). The imputation model included all study variables. Based on the percentage of missing data, we produced 10 imputed datasets and used Rubin's rules to obtain the final imputed estimates of the parameters of interest. Results using multiply imputed data were reported for all association analyses in the main text.

### 2.5 Sensitivity analyses

We also conducted sensitivity analyses to cheque the robustness of our findings using each indicator of health-compromising behaviours (cigarette smoking, alcohol drinking, and physical inactivity) and health service use (emergency service use, general practitioner service use, hospitalisation, and patient-provider communication).

## 3 Results

### 3.1 Participants' sociodemographics

In total, 7,264 teaching staff completed the survey, with a response rate of 93.9% (7,264/7,738). The average age of participants was 35.7 ± 8.3 years (age range: 18–68). Most participants were female (83.8%), from Han ethnicity (98.4%), and from married families (74.0%). The top three subjects of teaching were literacy (26.2%), math (25.8%) and English (11.1%) (see Table 2).

**Table 2:** Summary of participants' characteristics and distribution of health literacy and health outcomes.

**Participants' characteristics**	**Frequency (%)**
**Geographic location**	
Jingkai District	1,257 (17.3)
Zhongmou County	6,007 (82.7)
**School type**	
Primary school	4,836 (66.6)
Secondary school	2,428 (33.4)
**Sex**	
Female	6,086 (83.8)
Male	1,178 (16.2)
**Age group**	
30 years or below	2,448 (33.8)
31–39 years	2,609 (36.1)
40 years or above	2,178 (30.1)
**Ethnicity**	
Han	7,150 (98.4)
Ethnic minorities	114 (1.6)
**Marital status**	
Unmarried	1,686 (23.2)
Married	5,378 (74.0)
Other^*^	200 (2.8)
**Education level**	
Bachelor or above	6,338 (87.3)
Diploma or below	926 (12.7)
**Teaching duration**	
1–4 years	2,343 (32.3)
5–9 years	1,891 (26.1)
10–14 years	731 (10.1)
15–19 years	534 (7.4)
20–24 years	816 (11.2)
25 years or more	939 (12.9)
**Teaching subject**	
Literacy	2,029 (26.2)
Math	1,994 (25.8)
English	859 (11.1)
Physics	183 (2.4)
Chemistry	107 (1.4)
Biology	131 (1.7)
History	193 (2.5)
Geography	117 (1.5)
Politics	252 (3.3)
Physical education	438 (5.7)
Music	264 (3.4)
Art	256 (3.3)
Health	38 (0.5)
Other	226 (2.9)
More than one subject	651 (8.4)
**Health awareness in daily life**	
Very important	7,168 (98.7)
Not very important	96 (1.3)
**Chronic health conditions**	
No	6,050 (83.3)
Yes	1,214 (16.7)
**Medical insurance**	
Medical insurance for urban workers	5,365 (74.8)
Medical insurance for urban and rural residents	456 (6.4)
Rural cooperative medical insurance	1,272 (17.7)
Commercial medical insurance	80 (1.1)
**Personal health literacy**	
Excellent	901 (13.2)
Sufficient	2,046 (30.0)
Problematic	2,942 (43.1)
Inadequate	942 (13.8)
**School health literacy environment**	
Supportive	2,217 (31.0)
Not supportive	4,931 (69.0)
**Health status**	
Good	6,178 (85.0)
Poor	1,086 (15.0)
**At least one health-compromising behaviour**	
No	1,799 (24.8)
Yes	5,465 (75.2)
**At least one health service use**	
No	1,271 (17.6)
Yes	5,949 (82.4)
**Healthcare cost**	
<1,000 RMB	2,847 (39.3)
≥1,000 RMB	4,396 (60.7)

Observed data are shown (*n* = 7,264).

^*^The category of “other” includes those who are divorced or widowed.

### 3.2 Distribution of health literacy and health outcomes

Overall, schoolteachers had an average score of 75.17 ± 25.97 for personal health literacy and scored school health literacy environments with 25.30 ± 6.35. We found 43.1% and 13.8% of teachers had problematic and inadequate health literacy, respectively (see Table 2). Only one third (31.0%) of schoolteachers perceived their schools had supportive health literacy environments. 15.0% of schoolteachers had poor health status, 75.2% had at least one health-compromising behaviour, 82.4% had at least one health service use, and 60.7% spent 1,000 RMB or more for the out-of-pocket health expenditure.

### 3.3 Distribution of health literacy by participants' characteristics

Table 3 shows the distribution of personal health literacy and school health literacy environments by participants' characteristics. Schoolteachers had high personal health literacy scores if they were from primary schools, female, younger, unmarried, taught physical education, had high health awareness in daily life, had no chronic health conditions, and had medical insurance for urban and rural residents. Similarly, schoolteachers perceived high levels of school health literacy environments if they were from primary schools, younger, from Han ethnicity backgrounds, unmarried, taught physical education, had high health awareness in daily life, had no chronic health conditions, and had medical insurance for urban and rural residents.

**Table 3:** Distribution of health literacy by participants' characteristics, using imputed samples (*n* = 7,264).

**Participants' characteristics**	**Mean(**±**SD)**	
	**Personal health literacy**	**School health literacy environment**
**Geographic location**		
Jingkai District	73.47 (72.00, 74.93)^a^	25.50 (25.15, 25.85)^a^
Zhongmou County	74.67 (73.98, 75.35)^a^	25.21 (25.05, 25.38)^a^
**School type**		
Primary school	75.49 (74.75, 76.22)^a^	25.73 (25.56, 25.90)^a^
Secondary school	72.42 (71.30, 73.54) ^b^	24.33 (24.06, 24.60)^b^
**Sex**		
Female	74.61 (73.96, 75.25)^a^	25.29 (25.13, 25.45)^a^
Male	73.70 (71.96, 75.44)^a^	25.14 (24.77, 25.52)^a^
**Age group**		
30 years or below	80.48 (79.55, 81.42)^a^	26.34 (26.10, 26.57)^a^
31–39 years	74.48 (73.47, 75.49) ^b^	25.40 (25.15, 25.64)^b^
40 years or above	67.69 (66.45, 68.92) ^c^	23.90 (23.62, 24.19)^c^
**Ethnicity**		
Han	74.51 (73.89, 75.13) ^a^	25.28 (25.14, 25.43)^a^
Ethnic minorities	71.24 (65.84, 76.64) ^a^	23.96 (22.56, 25.35)^b^
**Marital status**		
Unmarried	79.23 (78.04, 80.42) ^a^	25.97 (25.69, 26.26)^a^
Married	73.11 (72.38, 73.83) ^b^	25.05 (24.87, 25.22)^b^
Other^*^	70.66 (66.67, 74.66) ^b^	25.08 (24.12, 26.04)^ab^
**Education level**		
Bachelor or above	74.43 (73.77, 75.09)^a^	25.20 (25.04, 25.36)^a^
Diploma or below	74.67 (72.86, 76.49)^a^	25.70 (25.31, 26.09)^a^
**Teaching duration**		
1-4 years	79.98 (79.01, 80.94)^a^	26.57 (26.34, 26.79)^a^
5-9 years	76.13 (74.93, 77.33)^b^	25.37 (25.07, 25.66)^b^
10-14 years	73.29 (71.31, 75.26)^c^	24.80 (24.32, 25.29)^c^
15-19 years	71.57 (69.12, 74.02)^c^	24.23 (23.65, 24.81)^cd^
20-24 years	68.58 (66.61, 70.54)^d^	24.36 (23.91, 24.82)^cd^
25 years or more	65.06 (63.19, 66.93) ^e^	23.54 (23.10, 23.98)^e^
**Teaching subject**		
Literacy	75.02 (73.84, 76.21)^ac^	25.28 (25.00, 25.56)^ace^
Math	73.36 (72.16, 74.56)^ad^	25.39 (25.12, 25.66)^c^
English	73.92 (72.07, 75.76)^ad^	24.82 (24.35, 25.29) ^ab^
Physics	66.50 (62.12, 70.88)^b^	23.81 (22.82, 24.80)^bgf^
Chemistry	68.39 (62.81, 73.98)^bdf^	23.00 (21.58, 24.43)^g^
Biology	76.13 (71.46, 80.80)^ag^	26.04 (25.15, 26.93)^ceh^
History	72.35 (68.12, 76.59)^af^	24.02 (23.03, 25.01)^bg^
Geography	74.80 (70.23, 79.38)^afi^	24.33 (23.07, 25.59)^acfg^
Politics	73.18 (69.53, 76.83)^afj^	25.03 (24.16, 25.90)^acf^
Physical education	82.47 (79.98, 84.96)^ek^	27.05 (26.50, 27.61)^dh^
Music	79.24 (76.00, 82.49)^gikl^	26.53 (25.68, 27.39)^dhi^
Art	76.41 (72.89, 79.93)^hijm^	25.83 (24.98, 26.67)^cei^
Health	72.38 (61.05, 83.70)^ablm^	24.56 (21.87, 27.25)^acfgi^
Other	74.05 (70.02, 78.08)^aflm^	25.51 (24.49, 26.53)^aci^
More than one subject	73.40 (71.25, 75.55)^afm^	24.80 (24.27, 25.32)^acf^
**Health awareness in daily life**		
Very important	74.67 (74.05, 75.28)^a^	25.30 (25.16, 25.45)^a^
Not very important	59.03 (52.30, 65.76)^b^	22.23 (20.74, 23.71)^b^
**Chronic health conditions**		
No	76.96 (76.33, 77.58)^a^	25.79 (25.64, 25.95)^a^
Yes	62.02 (60.25, 63.78)^b^	22.63 (22.24, 23.03)^b^
**Medical insurance**		
Medical insurance for urban workers	73.77 (73.06, 74.48)^a^	24.98 (24.80, 25.15)^a^
Medical insurance for urban and rural residents	79.28 (76.79, 81.77)^b^	26.55 (26.03, 27.07)^b^
Rural cooperative medical insurance	75.49 (73.99, 76.99)^c^	26.01 (25.68, 26.34)^bc^
Commercial medical insurance	76.64 (70.87, 82.42)^abc^	25.15 (23.62, 26.68)^abc^

SD, standard deviation. The category of “other” includes those who are divorced or widowed. Distribution of health literacy with the same superscript letter indicates no statistical difference between groups.

### 3.4 Associations between health literacy with health outcomes

Compared with those who had excellent health literacy, schoolteachers with inadequate health literacy had higher odds of poor health status [odds ratio (OR) = 5.79, 95% CI = 3.84, 8.73], at least one health-compromising behaviour (OR = 2.90, 95% CI = 2.29, 3.68), at least one health service use (OR = 2.73, 95% CI = 2.07, 3.61), and more healthcare cost (OR = 2.51, 95% CI = 2.00, 3.16), after adjusting for all covariates and school health literacy environments (see Table 4). Similarly, schoolteachers who perceived their school had low levels of school health literacy environments had higher odds of poor health status (OR = 1.62, 95% CI = 1.32, 1.99), at least one health-compromising behaviour (OR = 1.39, 95% CI = 1.22, 1.58), at least one health service use (OR = 1.78, 95% CI = 1.54, 2.06), and more healthcare cost (OR = 1.13, 95% CI = 1.00, 1.27), after adjusting for all covariates and personal health literacy.

**Table 4:** Associations between health literacy and health outcomes, using imputed samples (*n* = 7,264).

	**Model 1**	**Model 2**	**Model 3**
***Association with poor health status***			
**Personal health literacy**			
Excellent	Ref	Ref	Ref
Sufficient	1.94 (1.30, 2.91)	1.69 (1.12, 2.54)	1.39 (0.91, 2.11)
Problematic	5.30 (3.67, 7.64)	3.93 (2.71, 5.69)	3.07 (2.08, 4.51)
Inadequate	12.36 (8.46, 18.07)	7.73 (5.21, 11.46)	5.79 (3.84, 8.73)
**School health literacy environment**			
Supportive	Ref	Ref	Ref
Not supportive	3.11 (2.59, 3.73)	2.36 (1.95, 2.86)	1.62 (1.32, 1.99)
***Association with at least one health-compromising behaviour***			
**Personal health literacy**			
Excellent	Ref	Ref	Ref
Sufficient	1.87 (1.59, 2.20)	1.93 (1.63, 2.28)	1.71 (1.43, 2.04)
Problematic	2.50 (2.13, 2.93)	2.81 (2.38, 3.32)	2.38 (1.99, 2.85)
Inadequate	3.08 (2.50, 3.79)	3.55 (2.84, 4.43)	2.90 (2.29, 3.68)
**School health literacy environment**			
Supportive	Ref	Ref	Ref
Not supportive	1.71 (1.53, 1.91)	1.80 (1.61, 2.03)	1.39 (1.22, 1.58)
***Association with at least one health service use***			
**Personal health literacy**			
Excellent	Ref	Ref	Ref
Sufficient	2.25 (1.88, 2.68)	2.12 (1.77, 2.54)	1.72 (1.42, 2.08)
Problematic	3.58 (3.01, 4.25)	3.07 (2.57, 3.67)	2.30 (1.90, 2.80)
Inadequate	5.00 (3.90, 6.42)	3.93 (3.02, 5.10)	2.73 (2.07, 3.61)
**School health literacy environment**			
Supportive	Ref	Ref	Ref
Not supportive	2.55 (2.25, 2.89)	2.31 (2.02, 2.63)	1.78 (1.54, 2.06)
***Association with healthcare cost more than 1,000 RMB***			
**Personal health literacy**			
Excellent	Ref	Ref	Ref
Sufficient	1.41 (1.20, 1.65)	1.31 (1.11, 1.55)	1.25 (1.05, 1.49)
Problematic	2.04 (1.75, 2.37)	1.64 (1.40, 1.93)	1.55 (1.30, 1.84)
Inadequate	3.67 (3.00, 4.49)	2.71 (2.18, 3.36)	2.51 (2.00, 3.16)
**School health literacy environment**			
Supportive	Ref	Ref	Ref
Not supportive	1.58 (1.43, 1.75)	1.35 (1.21, 1.50)	1.13 (1.00, 1.27)

Model 1: Unadjusted; Model 2: Adjusted for geographic location, school type, sex, age group, ethnicity, marital status, education level, teaching duration, teaching subject, health awareness in daily life, chronic health conditions, and medical insurance; Model 3: Additionally adjusted for school health literacy environment when examining the impact of personal health literacy or additionally adjusted for personal health literacy when examining the impact of school health literacy environment.

We also found similar results when examining the associations of personal health literacy and school health literacy environments with each indicator of health-compromising behaviours (see Appendix 2) and health service use (see Appendix 3).

## 4 Discussion

### 4.1 Main findings of this study

Using a cross-sectional study design, we examined the relationships between personal health literacy, school health literacy environments, and a range of health outcomes among Chinese schoolteachers. This was the first study in Asia to assess organisational health literacy in school settings. Specifically, we had three main findings: (i) there was a high proportion (56.9%) of schoolteachers with inadequate or problematic health literacy; (ii) there were clear sociodemographic differences (e.g., age, marital status, school type) in schoolteacher's personal health literacy and school health literacy environments; (iii) Both personal health literacy and school health literacy environments were associated with health status, health-compromising behaviours, health service use, and healthcare cost.

Consistent with findings from previous research (20, 22), we found that low health literacy was prevalent (56.9%) in our sample when using the HLS_19_-Q12. The 2012 Chinese national health literacy survey found that 81.5% of Chinese schoolteachers had low health literacy (23). Internationally, Yilmazel and Cetinkaya (20) used the 6-item Newest Vital Sign to measure 500 primary and secondary schoolteachers' health literacy in Turkey and found that 73.8% of teachers had low health literacy. Denuwara and Gunawardena (22) found that 32.5% of secondary teachers had low health literacy in Sri Lanka when using the 47-item European Health Literacy Survey Questionnaire, whereas Rahimi and Elahe (25) found 48.3%−60% of primary teachers had low health literacy in Iran when using the 36-item Test of Functional Health Literacy in Adults. While these studies use different instruments to measure health literacy, there is consistent evidence on the high prevalence of low health literacy among schoolteachers, indicating the pressing need to improve their health literacy.

We found that schoolteachers' health literacy levels varied by school type, age group, marital status, teaching duration, teaching subject, health awareness in daily life, chronic health condition status, and medical insurance type. Most of these findings align with previous similar studies (20, 22), showing that schoolteachers tended to have low levels of health literacy if they were older, had longer teaching duration, and had chronic health conditions. This highlights that future intervention studies should consider these characteristics when targeting schoolteachers' health literacy. However, we did not find differences in health literacy by geographic location, sex, and highest educational level, which was contrary to previous research (20, 21). One possible reason could be the convenience sampling approach, in which we recruited schoolteachers from two districts in one city, thus contributing to the homogeneity of our samples (e.g., 87.3% had a Bachelor's degree or above). Future research is needed to use more representative samples to investigate these influencing factors of schoolteachers' health literacy.

Compared to previous studies that examined influencing factors of schoolteachers' health literacy (20–22, 25), we added evidence on the association between personal health literacy and health outcomes. We found teachers with lower health literacy were likely to have poorer health status, more health-compromising behaviours, more health service use, and more healthcare costs. These findings are consistent with previous systematic reviews (2, 26) and other population-based studies (27, 28). The potential pathways linking personal health literacy with health outcomes include both personal factors (e.g., self-efficacy, health knowledge) and system factors (e.g., social support, provider competence) (29). Our findings suggest promoting schoolteachers' health literacy skills has the potential to improve their health outcomes. As shown in the recent evidence base (30, 31), health literacy interventions can lead to improved health outcomes in the general population. Future experimental research is needed among schoolteachers to evaluate the effectiveness of tailored health literacy interventions to improve their health and wellbeing.

The present study also extends the current literature by examining the school health literacy environment. In keeping with the distribution of personal health literacy, we found a similar pattern of school health literacy environments. Teachers perceived lower levels of school health literacy environments if they were older, from ethnic minority backgrounds, married, had longer teaching duration, taught subjects other than physical education and biology, had low health awareness, had chronic health conditions, and had medical insurance for urban workers. We also found lower levels of school health literacy environments were associated with poor health status, more health-compromising behaviours, more health service use, and more healthcare costs among teachers. Aligning with previous HPS research (16, 17), we found that school health literacy environments were an important situational factor in influencing schoolteachers' health and wellbeing, with potential direct and indirect pathways through personal health literacy (5), social support (16), and mental health (32).

### 4.2 Limitations of this study

There were several limitations that should be noted. First, we used convenience sampling to recruit schoolteachers from two districts of Zhengzhou, Henan Province. Our findings may not generalise to other geographic regions or populations. Future research is needed to recruit more representative samples to replicate our findings. Second, measurement errors may exist for self-report instruments. In the present study, we used previously validated items or instruments to enhance the validity and reliability of key measures, thus minimising the self-report bias. It would be interesting in future research to use objective instruments (e.g., Newest Vital Sign) to measure health literacy and compare whether results are consistent between objective and subjective instruments. Finally, our findings are based on the cross-sectional study design, therefore we could not establish causality. Further research using longitudinal or experimental designs is needed to confirm our findings.

### 4.3 Implications for future research and practise

Findings from the present study shed light on the critical relationships between personal health literacy, school health literacy environments, and health outcomes among Chinese schoolteachers. Future research may consider using similar approaches to examining the role of personal health literacy and health literacy environments in other populations (e.g., children, older adults, patients) or settings (e.g., workplaces, hospitals, communities). It would be also worthwhile to explore whether personal health literacy interacts with health literacy environments to contribute to health outcomes in future.

We found that more than half of schoolteachers had inadequate or problematic health literacy. It is imperative for governments and schools to design and implement interventions to improve schoolteachers' health literacy. To improve health outcomes for schoolteachers, both educational programs and organisational change are needed to improve personal health literacy and school environments. Findings from a recent systematic review show that educational and motivational health literacy interventions with different strategies (e.g., websites, leaflets, smartphone apps) are effective to improve health outcomes (33). Recently, there has been increasing attention to school-based programs that aim to promote schoolteachers' health literacy and improve their health outcomes. The *HeLit-Schools* (14) and *HealthLit4Kids* (34) are two successful programs that highlight the need for organisational change to create supportive environments that foster health literacy. For example, the *HeLit-Schools* (14) program encourages schools to become health literate, not just by delivering health education to students, but by aligning school structures, communication, and leadership with health literacy goals. This includes, but not limited to, integrating health literacy into school planning documents and policies, providing professional development to enhance schoolteachers' health literacy, and empowering students through participatory activities. Only through a whole-of-school approach can a supportive environment be created that improves the wellbeing of both schoolteachers and children.

## 5 Conclusion

This study examined the relationship between health literacy and schoolteachers' health outcomes from two aspects: personal health literacy and school health literacy environments. Our findings highlight that both personal health literacy and school health literacy environments are important to schoolteachers' health outcomes. Promoting health literacy at both individual and organisational levels has the potential to improve population health and reduce health disparities.

## Data Availability

The raw data supporting the conclusions of this article will be made available by the authors, without undue reservation.
