# Supplementary material for: The impact of personal health literacy and school health literacy environments on schoolteachers' health outcomes
Source: Front Public Health. 2025 May 15;13:1570615. doi: 10.3389/fpubh.2025.1570615 (PMC12119273; doi:10.3389/fpubh.2025.1570615)
Supplement: Supplementary file 1 [file Table_1.docx]

Supplementary Material

**Appendix 1.** Missing data across key study variables (n=7264).

| **Participants' characteristics** | **Frequency (%)** |
| --- | --- |
| Geographic location | 0 |
| School type | 0 |
| Sex | 0 |
| Age group | 29 (0.4) |
| Ethnicity | 0 |
| Marital status | 0 |
| Education level | 0 |
| Duration of teaching | 13 (0.2) |
| Subject of teaching | 0 |
| Health awareness in daily life | 0 |
| Chronic health conditions | 0 |
| Medical insurance | 96 (1.2) |
| Global health status | 0 |
| Cigarette smoking | 0 |
| Alcohol drinking | 0 |
| Physical inactivity | 0 |
| At least one health-compromising behaviour | 0 |
| Emergency service use | 101 (1.3) |
| General practitioner service use | 77 (1.0) |
| Hospitalisation | 72 (0.9) |
| Patient-provider communication | 97 (1.3) |
| At least one health service use | 45 (0.6) |
| Healthcare coast | 22 (0.3) |
| Personal health literacy | 447 (5.8) |
| School health literacy environment | 1.5) |

**Appendix 2.** Associations between personal health literacy, school health literacy environment and each indicator of health behaviours, using imputed samples (n=7264).

|  | **Model 1** | **Model 2** | **Model 3** |
| --- | --- | --- | --- |
| ***Association with cigarette smoking*** | | | |
| Personal health literacy |  |  |  |
| Excellent | Ref | Ref | Ref |
| Sufficient | 0.84 (0.59, 1.19) | 1.00 (0.66, 1.51) | 1.14 (0.73, 1.78) |
| Problematic | 0.60 (0.43, 0.85) | 0.87 (0.57, 1.33) | 1.03 (0.64, 1.66) |
| Inadequate | 0.77 (0.51, 1.16) | 0.74 (0.45, 1.22) | 0.90 (0.52, 1.56) |
| School health literacy environment | |  |  |
| Supportive | Ref | Ref | Ref |
| Not supportive | 0.81 (0.63, 1.03) | 0.74 (0.55, 1.00) | 0.75 (0.53, 1.05) |
| ***Association with alcohol drinking*** | | | |
| Personal health literacy |  |  |  |
| Excellent | Ref | Ref | Ref |
| Sufficient | 1.09 (0.89, 1.33) | 1.39 (1.10, 1.75) | 1.27 (1.00, 1.62) |
| Problematic | 1.12 (0.93, 1.36) | 1.61 (1.29, 2.01) | 1.43 (1.12, 1.82) |
| Inadequate | 1.32 (1.06, 1.65) | 1.63 (1.24, 2.13) | 1.42 (1.06, 1.89) |
| School health literacy environment | |  |  |
| Supportive | Ref | Ref | Ref |
| Not supportive | 1.25 (1.10, 1.42) | 1.38 (1.19, 1.59) | 1.25 (1.06, 1.47) |
| ***Association with physical inactivity*** | | | |
| Personal health literacy |  |  |  |
| Excellent | Ref | Ref | Ref |
| Sufficient | 1.87 (1.60, 2.19) | 1.88 (1.59, 2.22) | 1.66 (1.40, 1.97) |
| Problematic | 2.63 (2.26, 3.06) | 2.85 (2.42, 3.36) | 2.40 (2.01, 2.86) |
| Inadequate | 2.98 (2.46, 3.60) | 3.69 (2.99, 4.55) | 3.00 (2.40, 3.75) |
| School health literacy environment | |  |  |
| Supportive | Ref | Ref | Ref |
| Not supportive | 1.67 (1.51, 1.85) | 1.83 (1.64, 2.05) | 1.41 (1.25, 1.60) |

Model 1: Unadjusted; Model 2: Adjusted for geographic location, school type, sex, age group, ethnicity, marital status, education level, duration of teaching, subject of teaching, health awareness in daily life, chronic health conditions, and medical insurance; Model 3: Additionally adjusted for school health literacy environment when examining the impact of personal health literacy or additionally adjusted for personal health literacy when examining the impact of school health literacy environment.

**Appendix 3.** Associations between personal health literacy, school health literacy environment and each indicator of health service use, using imputed samples (n=7264).

|  | **Model 1** | **Model 2** | **Model 3** |
| --- | --- | --- | --- |
| ***Association with emergency service use*** | | | |
| Personal health literacy |  |  |  |
| Excellent | Ref | Ref | Ref |
| Sufficient | 1.36 (1.08, 1.71) | 1.30 (1.03, 1.64) | 1.13 (0.89, 1.44) |
| Problematic | 1.51 (1.21, 1.87) | 1.32 (1.06, 1.65) | 1.10 (0.87, 1.39) |
| Inadequate | 2.51 (1.97, 3.19) | 1.88 (1.47, 2.42) | 1.52 (1.16, 1.99) |
| School health literacy environment | |  |  |
| Supportive | Ref | Ref | Ref |
| Not supportive | 1.75 (1.51, 2.02) | 1.52 (1.31, 1.77) | 1.42 (1.21, 1.67) |
| ***Association with general practitioner service use*** | | | |
| Personal health literacy |  |  |  |
| Excellent | Ref | Ref | Ref |
| Sufficient | 2.20 (1.86, 2.60) | 2.07 (1.74, 2.47) | 1.69 (1.41, 2.03) |
| Problematic | 3.35 (2.85, 3.94) | 2.87 (2.42, 3.40) | 2.17 (1.81, 2.60) |
| Inadequate | 4.49 (3.58, 5.64) | 3.51 (2.76, 4.46) | 2.47 (1.91, 3.19) |
| School health literacy environment | |  |  |
| Supportive | Ref | Ref | Ref |
| Not supportive | 2.45 (2.19, 2.76) | 2.21 (1.96, 2.49) | 1.75 (1.53, 2.00) |
| ***Association with hospitalisation*** | | | |
| Personal health literacy |  |  |  |
| Excellent | Ref | Ref | Ref |
| Sufficient | 1.37 (1.12, 1.67) | 1.28 (1.04, 1.57) | 1.14 (0.92, 1.41) |
| Problematic | 1.64 (1.36, 1.98) | 1.35 (1.10, 1.64) | 1.16 (0.94, 1.42) |
| Inadequate | 2.84 (2.28, 3.53) | 2.02 (1.61, 2.54) | 1.69 (1.33, 2.15) |
| School health literacy environment | |  |  |
| Supportive | Ref | Ref | Ref |
| Not supportive | 1.72 (1.52, 1.95) | 1.46 (1.28, 1.67) | 1.34 (1.17, 1.54) |
| ***Association with patient-provider communication*** | | | |
| Personal health literacy |  |  |  |
| Excellent | Ref | Ref | Ref |
| Sufficient | 2.13 (1.81, 2.51) | 2.01 (1.70, 2.38) | 1.66 (1.39, 1.98) |
| Problematic | 3.35 (2.86, 3.92) | 2.91 (2.47, 3.43) | 2.24 (1.88, 2.68) |
| Inadequate | 4.26 (3.40, 5.34) | 3.42 (2.69, 4.35) | 2.47 (1.92, 3.19) |
| School health literacy environment | |  |  |
| Supportive | Ref | Ref | Ref |
| Not supportive | 2.33 (2.09, 2.61) | 2.13 (1.90, 2.39) | 1.68 (1.48, 1.91) |

Model 1: Unadjusted; Model 2: Adjusted for geographic location, school type, sex, age group, ethnicity, marital status, education level, duration of teaching, subject of teaching, health awareness in daily life, chronic health conditions, and medical insurance; Model 3: Additionally adjusted for school health literacy environment when examining the impact of personal health literacy or additionally adjusted for personal health literacy when examining the impact of school health literacy environment.
